# Globetrotting strangles: the unbridled national and international transmission of *Streptococcus equi* between horses

**DOI:** 10.1099/mgen.0.000528

**Published:** 2021-03-08

**Authors:** Catriona Mitchell, Karen F. Steward, Amelia R. L. Charbonneau, Saoirse Walsh, Hayley Wilson, John F. Timoney, Ulli Wernery, Marina Joseph, David Craig, Kees van Maanen, Annelies Hoogkamer-van Gennep, Albertine Leon, Lucjan Witkowski, Magdalena Rzewuska, Ilona Stefańska, Monika Żychska, Gunther van Loon, Ray Cursons, Olivia Patty, Els Acke, James R. Gilkerson, Charles El-Hage, Joanne Allen, Hiroshi Bannai, Yuta Kinoshita, Hidekazu Niwa, Teótimo Becú, John Pringle, Bengt Guss, Reinhard Böse, Yvonne Abbott, Lisa Katz, Bernadette Leggett, Tom C. Buckley, Shlomo E. Blum, Fátima Cruz López, Ana Fernández Ros, Maria Cristina Marotti Campi, Silvia Preziuso, Carl Robinson, J. Richard Newton, Ellen Schofield, Ben Brooke, Mike Boursnell, Nicolas de Brauwere, Roxane Kirton, Charlotte K. Barton, Khalil Abudahab, Ben Taylor, Corin A. Yeats, Richard Goater, David M. Aanensen, Simon R. Harris, Julian Parkhill, Matthew T. G. Holden, Andrew S. Waller

**Affiliations:** ^1^​ Animal Health Trust, Newmarket, UK; ^2^​ Gluck Equine Research Center, Lexington, USA; ^3^​ Central Veterinary Research Laboratory, Dubai, UAE; ^4^​ Emirates Racing Authority, Dubai, UAE; ^5^​ Animal Health Service (GD), Deventer, The Netherlands; ^6^​ Labéo Frank Duncombe, Caen, France; ^7^​ Institute of Veterinary Medicine, Warsaw University of Life Sciences – SGGW, Warsaw, Poland; ^8^​ Ghent University, Merelbeke, Belgium; ^9^​ University of Waikato, Hamilton, New Zealand; ^10^​ Massey University, Palmerston North, New Zealand; ^11^​ University of Melbourne, Melbourne, Australia; ^12^​ Japan Racing Association, Tochigi, Japan; ^13^​ Clinica Equina, Buenos Aires, Argentina; ^14^​ Department of Biomedical Science and Veterinary Public Health, Swedish University of Agricultural Sciences, Uppsala, Sweden; ^15^​ Labor Dr Böse GmbH, Harsum, Germany; ^16^​ University College Dublin, Dublin, Ireland; ^17^​ Irish Equine Centre, Naas, Ireland; ^18^​ Kimron Veterinary Institute, Bet Dagan, Israel; ^19^​ Universidad Complutense, Madrid, Spain; ^20^​ Exopol, Zaragoza, Spain; ^21^​ Al Khalediah Equine Hospital, Riyadh, Saudi Arabia; ^22^​ University of Camerino, Camerino, Italy; ^23^​ Redwings Horse Sanctuary, Norwich, UK; ^24^​ Weatherford Equine Medical Centre, Weatherford, TX, USA; ^25^​ Big Data Institute, Li Ka Shing Centre for Health Information and Discovery, Nuffield Department of Medicine, University of Oxford, Oxford, UK; ^26^​ Centre for Genomic Pathogen Surveillance, Wellcome Trust Sanger Institute, Cambridge, UK; ^27^​ University of Cambridge, Cambridge, UK; ^28^​ University of St Andrews, St Andrews, UK; ^29^​ Intervacc AB, Stockholm, Sweden; ^‡^​ Present address: Technology Networks, Sudbury, UK; ^§^​ Present address: Xampla, Cambridge, UK; ^#^​ Present address: University of Berlin, Berlin, Germany; ^¶^​ Present address: University of Cambridge, Cambridge, UK; ^**^​ Present address: University of Cambridge, Cambridge, UK; ^††^​ Present address: Royal Society for the Prevention of Cruelty to Animals, Horsham, UK; ^‡‡^​ Present address: Microbiotica Limited, Cambridge, UK

**Keywords:** genome diversity, pandemic, strangles, *Streptococcus equi*, transmission

## Abstract

The equine disease strangles, which is characterized by the formation of abscesses in the lymph nodes of the head and neck, is one of the most frequently diagnosed infectious diseases of horses around the world. The causal agent, *Streptococcus equi* subspecies *equi *, establishes a persistent infection in approximately 10 % of animals that recover from the acute disease. Such ‘carrier’ animals appear healthy and are rarely identified during routine veterinary examinations pre-purchase or transit, but can transmit *S. equi* to naïve animals initiating new episodes of disease. Here, we report the analysis and visualization of phylogenomic and epidemiological data for 670 isolates of *S. equi* recovered from 19 different countries using a new core-genome multilocus sequence typing (cgMLST) web bioresource. Genetic relationships among all 670 S. *equi* isolates were determined at high resolution, revealing national and international transmission events that drive this endemic disease in horse populations throughout the world. Our data argue for the recognition of the international importance of strangles by the Office International des Épizooties to highlight the health, welfare and economic cost of this disease. The Pathogenwatch cgMLST web bioresource described herein is available for tailored genomic analysis of populations of *S. equi* and its close relative *S. equi* subspecies *zooepidemicus* that are recovered from horses and other animals, including humans, throughout the world. This article contains data hosted by Microreact.

## Data Summary

The Illumina sequences generated and used in this study have been deposited at the National Center for Biotechnology Information (NCBI) under the BioProject study accession number PRJEB38019. The accession numbers for the sequences of each isolate are listed in Table S1 (available with the online version of this article). The Pathogenwatch web bioresource can be accessed at: https://pathogen.watch/collection/j8c022xfgu8v-globetrotting. Data can be visualized in Microreact at: https://microreact.org/project/mu0Al6YdZ.

**Significance as a Bioresource to the Community**

The mobility of populations of animals facilitates the transmission of pathogens throughout the world, with important health and economic consequences. We provide a new online Pathogenwatch bioresource and describe the population structure of a global collection of 670 isolates of *Streptococcus equi *, the causative agent of strangles in horses. Strangles causes significant health, welfare and economic cost to the equine industry, and is endemic throughout the world. Individual outbreaks can involve hundreds of animals, the disease kills approximately 2 % of those infected and the cost of some individual outbreaks can exceed £300 000. Using Pathogenwatch, we identify clear examples of the spread and transmission of *S. equi* at both national and international levels. Our data show that the transmission of *S. equi* is of international importance and that the identification of horses that are infected with *S. equi* pre- or immediately post-transit is likely to yield significant benefits to the global equine industry. We also report an ongoing population replacement event of importance for the diagnosis and prevention of this disease throughout the world. The Pathogenwatch bioresource is available for the determination and visualization of the population structure of *S. equi* and also its close relative *S. equi* subspecies *zooepidemicus *, which causes disease in a wide range of animals including horses, dogs, pigs, sheep, cattle and humans.

## Introduction

Strangles, caused by *Streptococcus equi* subspecies *equi *, is one of the most prevalent diseases of horses around the world [1]. The consequences of strangles can be serious, with some individual outbreaks involving hundreds of horses leading to associated costs exceeding £300 000 [2]. Only the geographically isolated population of horses in Iceland remains free of *S. equi *, which is believed to be due to a self-imposed ban on the import of horses that has been in place for over a thousand years [3]. Equine strangles is characterized by pyrexia, and the development of abscesses in the lymph nodes of the head and neck [4]. As the disease progresses, abscesses in the retropharyngeal lymph nodes rupture and drain into the guttural pouches, and then into the environment via the nasopharynx. Incomplete drainage of abscess material from the guttural pouches results in a proportion of convalescent animals becoming persistently infected with *S. equi *. These outwardly healthy ‘carrier’ animals can harbour *S. equi* for years, intermittently shedding bacteria into the environment where the organism can be taken up by naïve animals, triggering new outbreaks of disease [5–7].

*S. equi* was first identified in 1888 [8] and early studies found that restriction digestion of the DNA of different isolates yielded fragment profiles that were indistinguishable from one another by PFGE [9]. However, the analysis of the genomes of a collection of 225 contemporary isolates identified at least four genetically distinct clusters of *S. equi* and provided evidence that a global population replacement had occurred at the end of the 19th or beginning of the 20th century [10]. During this period of history, the international transport and mixing of horses that accompanied a series of global conflicts is proposed to have led to the emergence of a fitter strain of *S. equi* from which apparently all contemporary strains are derived [10]. Horses continue to be transported all over the world for trade, training and competition [11]. However, despite the global prevalence of strangles, the disease is conspicuously absent from the Office International des Épizooties (OIE) or World Organisation for Animal Health list of diseases, infections and infestations of the horse [https://www.oie.int/animal-health-in-the-world/oie-listed-diseases-2020/ (last accessed 22nd July 2020)], and no diagnostic testing for strangles is typically conducted pre-purchase, export or import of horses.

Routine molecular typing schemes, such as multilocus sequence typing (MLST), which is based on the sequence of fragments of seven genes, captures insufficient genetic diversity to adequately differentiate the population structure of *S. equi* and to resolve transmission events [10, 12]. A single locus typing scheme, based on variation of the DNA sequence encoding the N-terminal region of SeM, a novel M-like protein [13, 14], has previously been utilized to link the transmission of *S. equi* from the USA to Japan [13], and to the UK [15]. This sortase-processed cell surface protein binds to fibrinogen and immunoglobulin to impede the phagocytosis of *S. equi* by immune cells [16–19]. However, the utility of SeM typing as an epidemiological tool is limited by homoplasies [10] and the high rate of mutation of SeM, even within individual animals [10, 14, 20]. Here, we report the application of a novel web-based core-genome MLST (cgMLST) bioresource, implemented in Pathogenwatch, to rapidly determine the population structure of 670 isolates of *S. equi *, which were recovered from horses residing in 19 countries, for analysis and visualization alongside epidemiological data. We identify numerous examples of closely related strains of *S. equi* in geographically distant nations, highlighting that the lack of pre-export testing, used routinely for many animal diseases, facilitates the unbridled international transmission of *S. equi *.

## Methods

### Study collection

The origins and details of the 670 isolates of *S. equi* that were analysed in this study are listed in Table S1. The complete genome sequence of *Se*4047, which was isolated from a horse with strangles in the New Forest, Hampshire, UK, in 1990 was used as the reference genome in this study [21]. Two hundred and twenty-four assembled genomes of *S. equi* that had been generated to study the effects of persistent infection were also included [10]. The remaining 445 isolates were recovered from clinical samples submitted to the Animal Health Trust in the UK; the Gluck Equine Research Center in the USA; the Central Veterinary Research Laboratory in the United Arab Emirates (UAE); the Animal Health Service (GD) in the Netherlands; Labéo Frank Duncombe in France; the Institute of Veterinary Medicine, Warsaw University of Life Sciences – SGGW in Poland; Ghent University in Belgium; the University of Waikato and Massey University in New Zealand; the University of Melbourne in Australia; the Japan Racing Association in Japan; the Clinica Equina in Argentina; the Swedish University of Agricultural Sciences in Sweden; Labor Dr Böse GmbH in Germany; the Irish Equine Centre in Ireland; the Kimron Veterinary Institute in Israel; the Servico de Vigilancia Sanitaria Equina and Exopol in Spain; and the Al Khalediah Equine Hospital in Saudi Arabia. β-Haemolytic colonies of *S. equi* strains were recovered from glycerol stocks following overnight growth on COBA strep select plates (bioMérieux). Their identity was confirmed by a lack of fermentation of trehalose, ribose and sorbitol in purple broth (Becton Dickinson) [22]. Where appropriate and in order to maintain client confidentiality, isolates were assigned a geographical point of origin based on the submitting veterinary practice. An outbreak was defined as the recovery of at least one isolate with a unique geographical origin or from the same location, but separated by a time period of at least 6 months to account for some outbreaks requiring extensive efforts to identify and treat persistently infected horses.

### DNA preparation

A single colony of each of the 445 *S *. *equi* strains that were sequenced in this study was grown statically overnight in 3 ml Todd Hewitt (TH) broth containing 30 µg hyaluronidase ml^−1^ (Sigma) at 37 °C in a 5 % CO_2_ enriched atmosphere. Cultures were centrifuged, the pellet re-suspended in 200 µl GenElute Gram-positive lysis solution (Sigma) containing 250 units mutanolysin ml^−1^ and 2×10^6^ units lysozyme ml^−1^, and incubated for 1 h at 37 °C to allow efficient cell lysis. DNA was then purified using GenElute spin columns, according to the manufacturer’s instructions (Sigma).

### DNA sequencing and phylogenomic analysis

The previously assembled genomes of *S. equi* strain 4047 (*Se*4047) [21] and a further 224 strains of *S. equi* from the UK, Sweden, Ireland, Belgium, Canada, New Zealand, Australia, Saudi Arabia and the USA [10] were included in this study. For the remaining 445 strains of *S. equi *, library construction for Illumina sequencing was carried out as described previously [23]. DNA libraries were prepared using the Illumina Nextera XT DNA library preparation kit, according to the standard protocol (Illumina). The DNA libraries were pooled and quantified using the KAPA library quantification kit for Illumina platforms prior to sequencing on an Illumina MiSeq according to the manufacturer’s protocol. FastQ files were assembled using the Shovill assembly pipeline (https://github.com/tseemann/shovill). Genome assemblies for all 670 isolates were uploaded into the Pathogenwatch bioresource for *S. equi* (https://cgps.gitbook.io/pathogenwatch/), which assigns phylogeny based on the sequences of alleles in the core genome of *S. equi *. The collection in Pathogenwatch can be accessed at: https://pathogen.watch/collection/j8c022xfgu8v-globetrotting. The core genome of the *Se*4047 reference genome comprised a curated set of 1286 loci lacking mobile genetic elements (MGEs) (φSeq1, φSeq2, φSeq3, φSeq4, ICE*Se1* or ICE*Se2*), insertion sequences and sortase-processed proteins [10, 21]. Alleles of loci for which multiple copies are encoded within the *S. equi* genome, including *hasC1* and *hasC2*, were omitted also [21]. blast matches of the 1286 loci across each genome relative to the core genome of the *Se*4047 reference were extracted and aligned using mafft [24], and a database of the core-genome segments with a per cent identity was constructed. Hits below 80 % core-gene length or identity were removed as fragments. Each specific combination of substitutions within the core-genome loci relative to the *Se*4047 reference was assigned an allele. Indels were excluded from future analysis as they are often the result of assembly or sequencing errors [24]. The variant sites between each pair of assemblies were then used to reconstruct dendrograms using the ape package [25]. The resulting tree was midpoint rooted using the phangorn package [26]. Population structure was inferred using the population mixture analysis in BAPS v. 6.0 [27] by clustering of individuals with *k*=5, 5, 6, 6, 7, 7, 8, 8 based on the distribution of the 1286 alleles within the core genome of each isolate (Table S2). The phylogenetic reconstruction and associated metadata were then visualized using Microreact [28], which can be viewed at: https://microreact.org/project/mu0Al6YdZ. MLST and SeM-typing alleles were derived *in silico* from the WGS data using the BIGSdb software [12, 14, 29]. Homoplasies in the predicted amino acid sequences of SeM were identified using mega x [30]. Statistical analysis of the relationships between groups of isolates was performed using the Fisher’s exact two-sided test.

## Results

### Pathogenwatch facilitates the genetic differentiation of *S. equi* strains

**Fig. 1.**
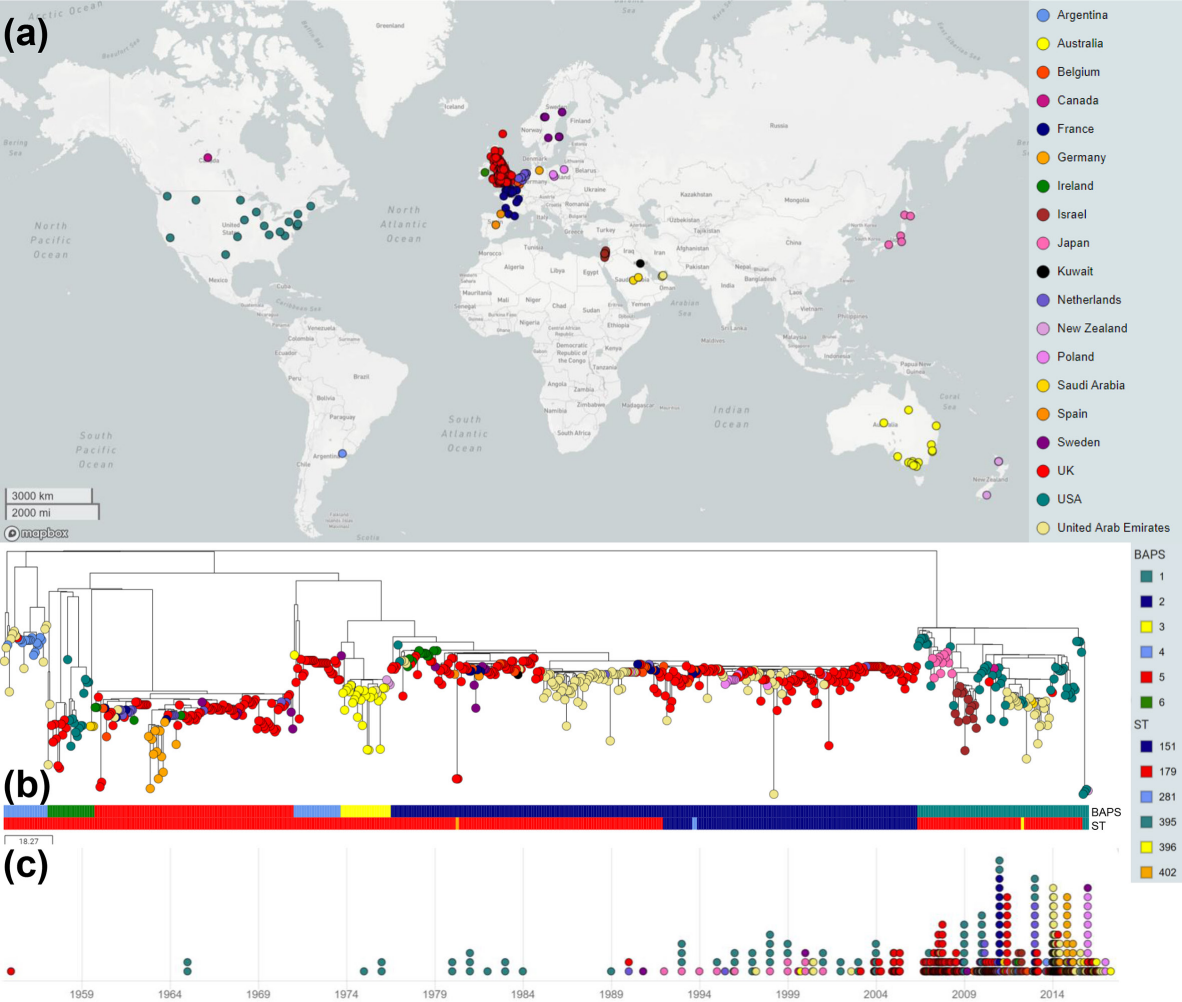
A global picture of *S. equi* diversity visualized in Microreact. Coloured circles indicate the country from which the isolates originated, as illustrated in the key. (**a**) World map depicting the country of origin of the 670 *S *. *equi* isolates. (**b**) Midpoint-rooted phylogenetic reconstruction of the *S. equi* population. The dendrogram was reconstructed from pairwise cgMLST scores using the ape package [25]. The resulting tree was midpoint rooted using the phangorn package [26]. The scale bar relates to horizontal branch lengths and indicates the number of cgSNPs that are proposed to have occurred on the vertical branches. The six genetically distinct BAPS clusters and six multilocus STs are indicated on the coloured metadata bars and associated key. (**c**) Timeline of the collection from 1955 to 2017.

Pathogenwatch was utilized to identify variant sites within a curated set of 1286 core-genome loci across 670 isolates of *S. equi* that were recovered from 19 countries between 1955 and 2017 (https://pathogen.watch/collection/j8c022xfgu8v-globetrotting). A midpoint-rooted phylogenetic reconstruction of the *S. equi* population based on the core-genome variant sites between each genome pair was then generated within Pathogenwatch and exported into Microreact for visualization of the genomic epidemiology (Fig. 1a, b, c, Tables S1 and S2; https://microreact.org/project/mu0Al6YdZ). Finally, the population structure of the collection, based on core-genome alleles assigned within Pathogenwatch, was inferred using BAPS [27]. Six clusters (BAPS-1 to BAPS-6) of genetically related strains were identified that contained 106, 325, 31, 56, 123 and 29 isolates, respectively (Fig. 1b). The isolates within each of these clusters differed from one another by, on average, between 13.3 (BAPS-2) to 73.2 (BAPS-6) pairwise core-genome SNPs (cgSNPs), whilst the mean number of pairwise cgSNPs between BAPS clusters in the collection was 90.9 (Tables 1 and S3, Fig. S1).

**Table 1.** Abundance and diversity of the *S. equi* population within each of the BAPS clusters

BAPS cluster	Total no. of genomes	Mean no. of pairwise cgSNPs (sd)
BAPS-1	102*	30.7 (12.5)
BAPS-2	325	13.3 (9.0)
BAPS-3	31	17.7 (11.4)
BAPS-4	56	52.1 (32.5)
BAPS-5	120†	14.5 (9.7)
BAPS-6	29	73.2 (39.0)
Intra-BAPS cluster	663‡	16.0 (13.8)
Inter-BAPS cluster	663	90.9 (17.8)

*The pairwise comparisons of the four Pinnacle IN isolates were excluded as this diversity reflects artificially induced variation.

†The pairwise comparisons of the three Equilis StrepE isolates were excluded as this diversity reflects artificially induced variation.

‡The pairwise comparisons of the four Pinnacle IN and three Equilis StrepE isolates were excluded as this diversity reflects artificially induced variation.

### MLST differentiates the *S. equi* collection into six sequence types (STs) within clonal complex 179 (CC-179)

All isolates were assigned to one of six different MLST STs using the standard MLST scheme in BIGSdb, all of which clustered into CC-179 [12]. The majority of isolates in the collection were ST-179 (*n*=505, 75 %) and these were distributed across all of the six BAPS clusters (Fig. 1b). ST-179 isolates were recovered from horses over the longest time period (1955 to 2017) and from all 19 countries in the collection. Four isolates of ST-395, a single locus variant (SLV) of ST-179, were identified in BAPS-1. ST-395 is restricted to the Pinnacle IN live attenuated vaccine, which is produced by Zoetis. The CF32 strain (named USACF32 in Pathogenwatch), which was used as the basis of the Pinnacle IN vaccine [31], was attenuated by mutagenic treatment with *N*-methyl-*N*′-nitro-*N*-nitrosoguanidine (NTG), generating 68 point mutations, including two SNPs in *spi* that convert the allele 45 of ST-179 to allele 69 [10, 32]. ST-395, therefore, represents an *in vitro* generated part of the MLST-defined population structure. Two isolates of ST-396, a SLV of ST-179, were recovered from two outbreaks of strangles (USA-13 and USA-33) in Kentucky and Pennsylvania in the USA during 2012, and clustered into BAPS-1. Another two isolates (UK121a and UK121b) that were recovered from the guttural pouches of the same healthy horse with a persistent *S. equi* infection in Leicestershire, UK, were also a SLV of ST-179, ST-402, and clustered into BAPS-2. The most frequently identified SLV of ST-179 was ST-151, which was identified in 154 (23 %) isolates of *S. equi* in the collection. ST-151 isolates were limited to BAPS-2 and were recovered from the UK, Belgium, the Netherlands, Poland and the UAE between 2005 and 2017. Finally, the three lone isolates of ST-281, a SLV of ST-151, were also limited to BAPS-2, and were recovered from two outbreaks of strangles (Fr-4 and Fr-13) near Paris, France, during 2011, and a horse in Belgium during 2012. Excluding 18 isolates for which no date of sampling was available, 223 (45 %) and 272 (55 %) isolates of ST-179, ST-395, ST-396 and ST-402 were recovered pre- and post-2010, respectively. In comparison, 19 (12 %) and 138 (88 %) isolates of ST-151 and its SLV, ST-281, were recovered from horses pre- and post-2010, respectively. ST-151 and ST-281 containing strains were significantly more likely to have been recovered from horses post-2010 (*P*<0.0001).

### Consensus SeM allele of each BAPS cluster is the most prevalent

The isolates in the collection possessed 103 different SeM alleles, excluding 13 isolates that contained deletions or insertions in SeM (Table S1). SeM alleles clustered in accordance with the Pathogenwatch phylogenetic reconstruction (Tables S1 and S4). Despite the large number of SeM alleles in the dataset, the consensus SeM alleles of each BAPS cluster were the most prevalent allele within that cluster. A total of 38 of 106 isolates (36 %) in BAPS-1 contained the SeM-28 allele, 223 of 325 isolates (69 %) in BAPS-2 contained the SeM-9 allele and 26 of 31 isolates (84 %) in BAPS-3 contained the SeM-100 allele. The strong immune selection pressure on SeM was highlighted by homoplasy where 25 SNPs arose independently in different BAPS clusters. These SNPs were predicted to lead to 20 different amino acid changes in SeM, including Glu^58^ to Asp, Val or Gly (Table S4).

### BAPS-1 is the predominant lineage present in the USA, but is shared with horses around the world

**Fig. 2.**
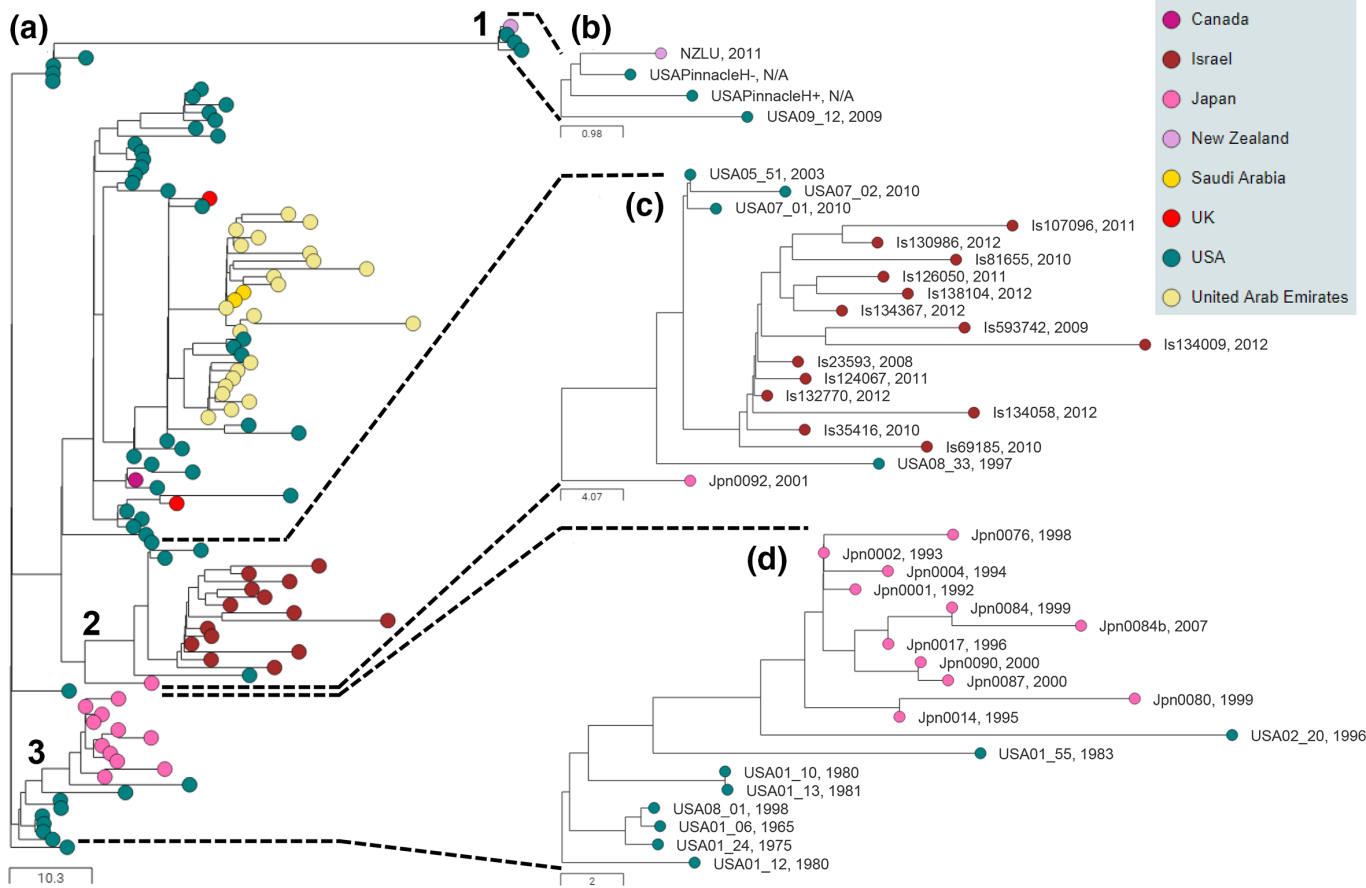
BAPS-1 illustrates international transmission events between the USA, UAE, Israel, Japan, Canada and the UK. Midpoint-rooted phylogenetic reconstruction of the *S. equi* population visualized in Microreact. The dendrogram was reconstructed from pairwise cgMLST scores using the ape package [25]. The resulting tree was midpoint rooted using the phangorn package [26]. The scale bars relate to horizontal branch lengths and indicate the number of cgSNPs that are proposed to have occurred on the horizontal branches. Coloured circles indicate the country from which the isolates originated, as indicated in the key. (**a**) The phylogenetic relationships of the 106 isolates in the BAPS-1 subtree. Three sub-groups are numbered. (**b**) Pinnacle IN vaccine strains in sub-group 1 and clinical isolates from New Zealand and Montana (USA). (**c**) Sub-group 2 contains isolate Jpn0092, and isolates from outbreaks in the USA and Israel. (**d**) Sub-group 3 contains isolates from outbreaks in the USA and Japan. Isolate names and year of isolation are shown. N/A, not applicable.

BAPS-1 contained 106 isolates, including 52 (76 %) of the 68 isolates recovered from horses in the USA, and all 14 and 12 isolates recovered from horses in Israel and Japan, respectively (Fig. 2a). The remaining isolates in BAPS-1 were recovered from horses in the UAE (*n*=22), Saudi Arabia (*n*=2), the UK (*n*=2), Canada (*n*=1) and New Zealand (*n*=1). The 24 isolates recovered from horses in the UAE and Saudi Arabia themselves clustered into two distinct groups (Fig. 2a). The first of these contained 16 isolates from seven outbreaks, UAE-9, UAE-10, UAE-11, UAE-21, UAE-22, UAE-23 and Sab-2, that occurred in the UAE and Saudi Arabia between 2013 and 2015. These isolates differed by a mean of 12 pairwise cgSNPs (range 1 to 40), suggesting that the outbreaks may have been caused by the import of a single horse to the UAE or Saudi Arabia. The other group of eight BAPS-1 isolates from the UAE were recovered from two outbreaks (UAE-7 and UAE-8) between October 2009 and February 2010, differed by a mean of 5 pairwise cgSNPs (range 1 to 10) (Table S3) and were likely caused by a different import event.

Sub-group 1 within BAPS-1 contained the haemolytic (H+) and non-haemolytic (H−) variants of the Pinnacle IN vaccine [10] (Fig. 2b). These clustered with isolate NZLU, which was recovered in 2011 from a horse with strangles in New Zealand, 1 month after it had been vaccinated with the Pinnacle IN vaccine [32], and USA09_12, which was recovered from a horse with strangles in Montana during 2009 with an unknown vaccination history. These isolates differed by between two and five pairwise cgSNPs relative to the Pinnacle vaccine strains (Table S3).

Sub-group 2 isolates of BAPS-1 were recovered from outbreaks of strangles in Maine, USA, during 2003 (USA-16), and Kentucky, USA, during 1997 (USA-7) and 2010 (USA-11), in addition to at least 11 outbreaks of strangles that occurred in Israel between 2008 and 2012 [33], differing by a mean of 18 pairwise cgSNPs (range 4 to 41) (Fig. 2c, Table S3). Finally, isolate Jpn0092 was recovered from a horse that developed strangles whilst in the Shiroi quarantine centre in Chiba, Japan, shortly after arrival from the USA [13].

Sub-group 3 isolates of BAPS-1 were recovered from 11 outbreaks of strangles in Japan between 1992 and 2007, which were linked to the import of a horse from Indiana, USA, in 1992, and differed by a mean of 7 pairwise cgSNPs (range 1 to 18) [13, 34] (Fig. 2d). These isolates were most closely related to an isolate from an outbreak of strangles in Kentucky, USA, during 1996 (USA-6), differing by between 16 and 26 pairwise cgSNPs (Table S3).

### BAPS-2 is the predominant genetic lineage in Europe and the Middle East

**Fig. 3.**
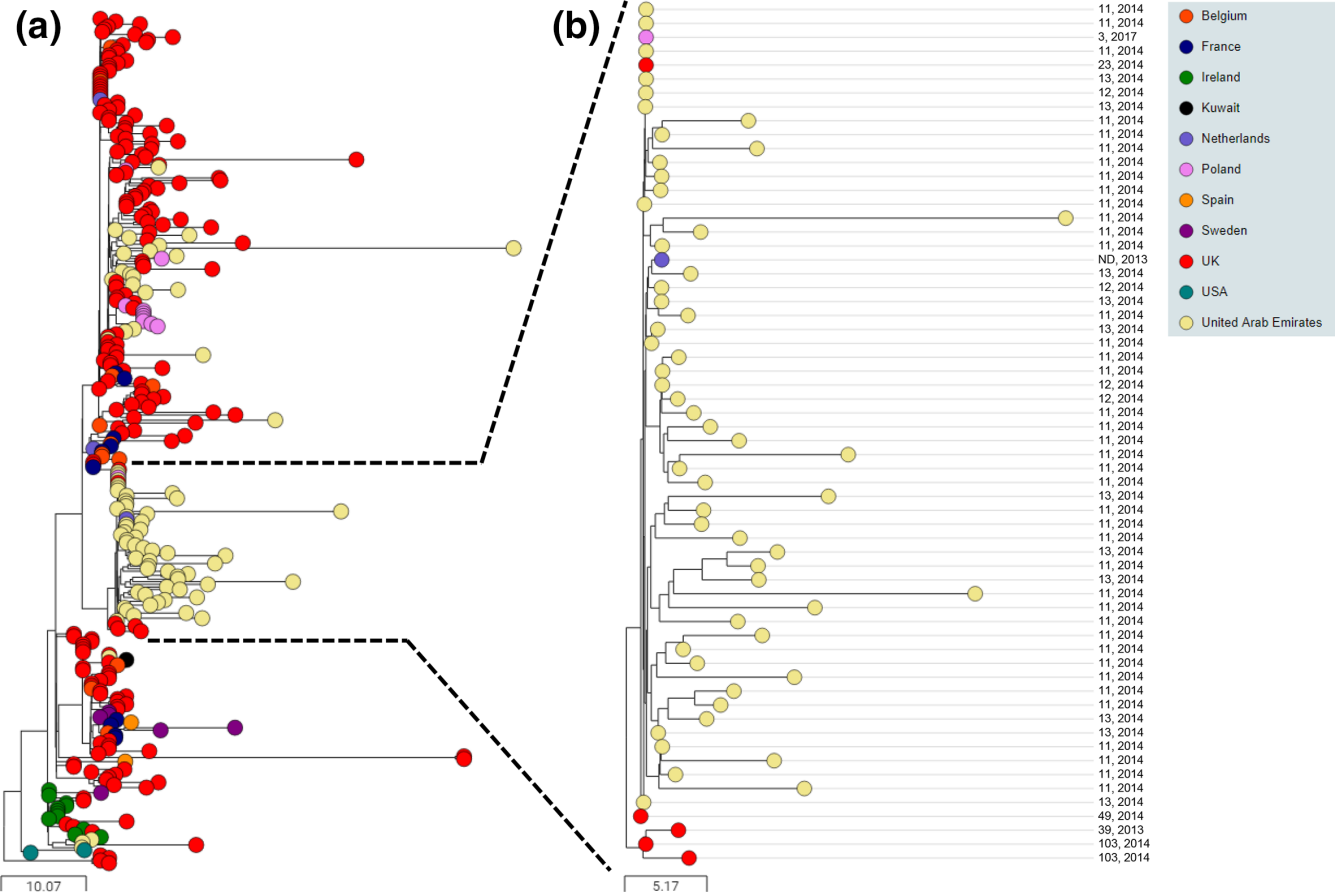
BAPS-2 is the dominant European cluster, with evidence of transmission between horses in the UAE. Midpoint-rooted phylogenetic reconstruction of the *S. equi* population visualized in Microreact. The dendrogram was reconstructed from pairwise cgMLST scores using the ape package [25]. The resulting tree was midpoint rooted using the phangorn package [26]. The scale bars relate to horizontal branch lengths and indicate the number of cgSNPs that are proposed to have occurred on the horizontal branches. Coloured circles indicate the country from which the isolates originated, as indicated in the key. (**a**) The phylogenetic relationships of the 325 isolates in the BAPS-2 cluster. (**b**) Phylogenetic relationships of isolates in one sub-group, which were recovered from three outbreaks of strangles in the UAE between January and June 2014, and cases in Poland, the UK and the Netherlands during 2017, April 2014 and 2013, respectively. The identity and year of the outbreaks from which the isolates were recovered is indicated on the right. nd, Not determined.

BAPS-2 contained 325 isolates, including 186 of 307 isolates (61 %) recovered from horses in the UK (Fig. 3a). BAPS-2 also included isolates recovered from horses in the UAE (*n*=80), Belgium (*n*=12), Ireland (*n*=12), Poland (*n*=11), France (*n*=10), Sweden (*n*=6), the Netherlands (*n*=3), Spain (*n*=2), the USA (*n*=2) and Kuwait (*n*=1). Overall, BAPS-2 accounted for 242 (60 %) of the 405 European isolates of *S. equi* in the collection and 81 (58 %) of 139 isolates from the Middle East. Further analysis of the UK population of *S. equi *, which was the most comprehensive of the countries sampled, identified that BAPS-2 strains were significantly more likely to be recovered between 2010 and 2016 [131 (77 %) of 170 isolates], than they were prior to 2010 [55 (40 %) of 137 isolates] (*P*<0.0001).

One branch of the BAPS-2 phylogeny was recovered predominantly from horses in the UAE that were involved in three outbreaks of strangles (Fig. 3b). A total of 40 of 67 isolates recovered from outbreak UAE-11 (between January and March 2014), all 4 isolates from outbreak UAE-12 (February 2014) and all 11 isolates from outbreak UAE-13 (between May and June 2014) clustered into this branch of BAPS-2, differing by a mean of 8 pairwise cgSNPs (range 0 to 44). Furthermore, an isolate from outbreak Pol-3 in Szamotuly, Poland, during 2017, outbreak UK-23 in Cheshire, UK, in April 2014, and an unnamed outbreak in the Netherlands during 2013 also clustered into this branch of the BAPS-2 phylogeny, sharing identity or differing by one pairwise cgSNP to several isolates from the outbreaks in the UAE (Fig. 3b, Table S3).

### BAPS-3 is the predominant genetic lineage in the Oceania region and is exchanged with horses in the UAE

**Fig. 4.**
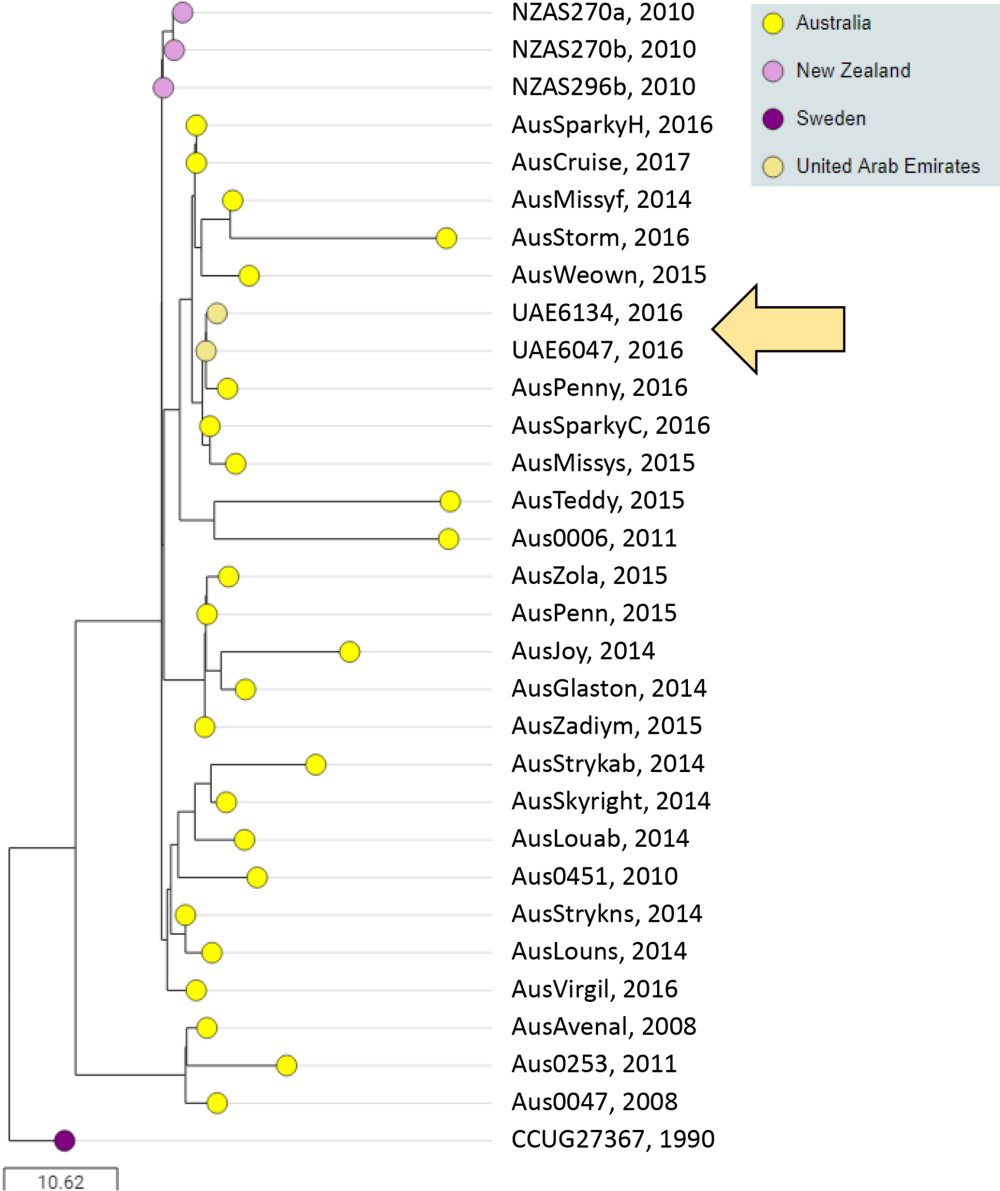
BAPS-3 is an Oceania cluster, exchanged with horses in the UAE. Midpoint-rooted phylogenetic reconstruction of the *S. equi* population visualized in Microreact. The dendrogram was reconstructed from pairwise cgMLST scores using the ape package [25]. The resulting tree was midpoint rooted using the phangorn package [26]. The scale bar relates to horizontal branch lengths and indicates the number of cgSNPs that are proposed to have occurred on the horizontal branches. Coloured circles indicate the country from which the isolates originated, as indicated in the key. The khaki arrow highlights the two isolates recovered from the UAE. Isolate names and year of isolation are shown.

BAPS-3 contained 31 isolates, including 25 (96 %) of 26 isolates recovered from horses in Australia (Fig. 4). BAPS-3 also contained three of the four isolates recovered from New Zealand, two isolates from the UAE and one isolate from Sweden. Isolates from Australia and New Zealand accounted for 90 % of isolates (28 of 31) in BAPS-3.

### BAPS-4 is limited to horses in Argentina, the UK and the UAE

**Fig. 5.**
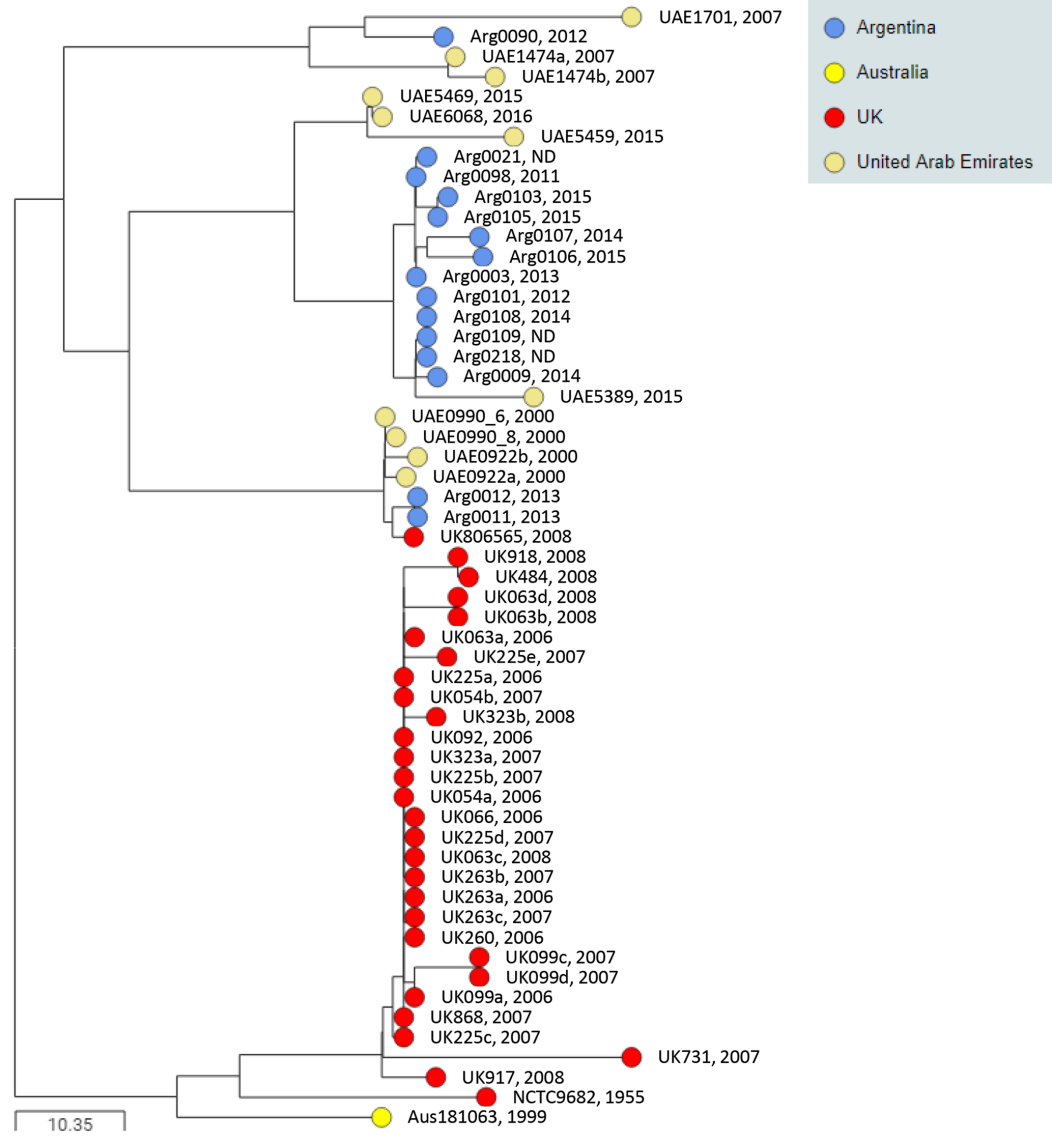
BAPS-4 illustrates the international transmission of *S. equi* between Argentina, the UAE and the UK. Midpoint-rooted phylogenetic reconstruction of the *S. equi* population visualized in Microreact. The dendrogram was reconstructed from pairwise cgMLST scores using the ape package [25]. The resulting tree was midpoint rooted using the phangorn package [26]. The scale bar relates to horizontal branch lengths and indicates the number of cgSNPs that are proposed to have occurred on the horizontal branches. Coloured circles indicate the country from which the isolates originated, as indicated in the key. Isolate names and year of isolation are shown. nd, Not determined.

The 56 isolates of BAPS-4 included all of the isolates recovered from Argentina (*n*=15), along with isolates from the UK (*n*=27 of 307), the UAE (*n*=11 of 119) and Australia (*n*=1 of 26) (Fig. 5). Isolate UK806565 was recovered from a polo pony in Surrey, UK, during 2008 and clustered with four isolates from outbreak UAE-2 during 2000, which also affected polo ponies, and two isolates from Argentina (Arg0011 and Arg0012) that together differed by a mean of 4 pairwise cgSNPs (range 0 to 6).

BAPS-4 also contained a branch of 29 isolates, 27 of which were recovered from a large outbreak of strangles (UK-91) that affected over 200 horses at an equine rescue centre in Lincolnshire, UK, between November 2006 and April 2008 (Fig. 5). These isolates differed by a mean of 6 pairwise cgSNPs (range 0 to 32). The most variable of these genomes was that of isolate UK731, which was recovered from a persistently infected carrier in November 2007 [10]. This branch included the NCTC 9682 strain, which was recovered from a horse in the UK in 1955, and an isolate recovered from a horse in Australia during 1999 [10].

The two branches in BAPS cluster 4 were paraphyletic, being located in two different positions in the phylogeny presented in Fig. 1(b) separated by BAPS clusters 5 and 6, and highlighted by the diversity of genomes within this cluster as shown in Fig. S1. Increased diversity within BAPS cluster 4 could be an effect of recombination, creating similarities between lineages that are picked up by the BAPS algorithm, but that are not represented in the consensus phylogenetic tree.

### BAPS-5 was the predominant European cluster of *S. equi *, but is becoming less prevalent

**Fig. 6.**
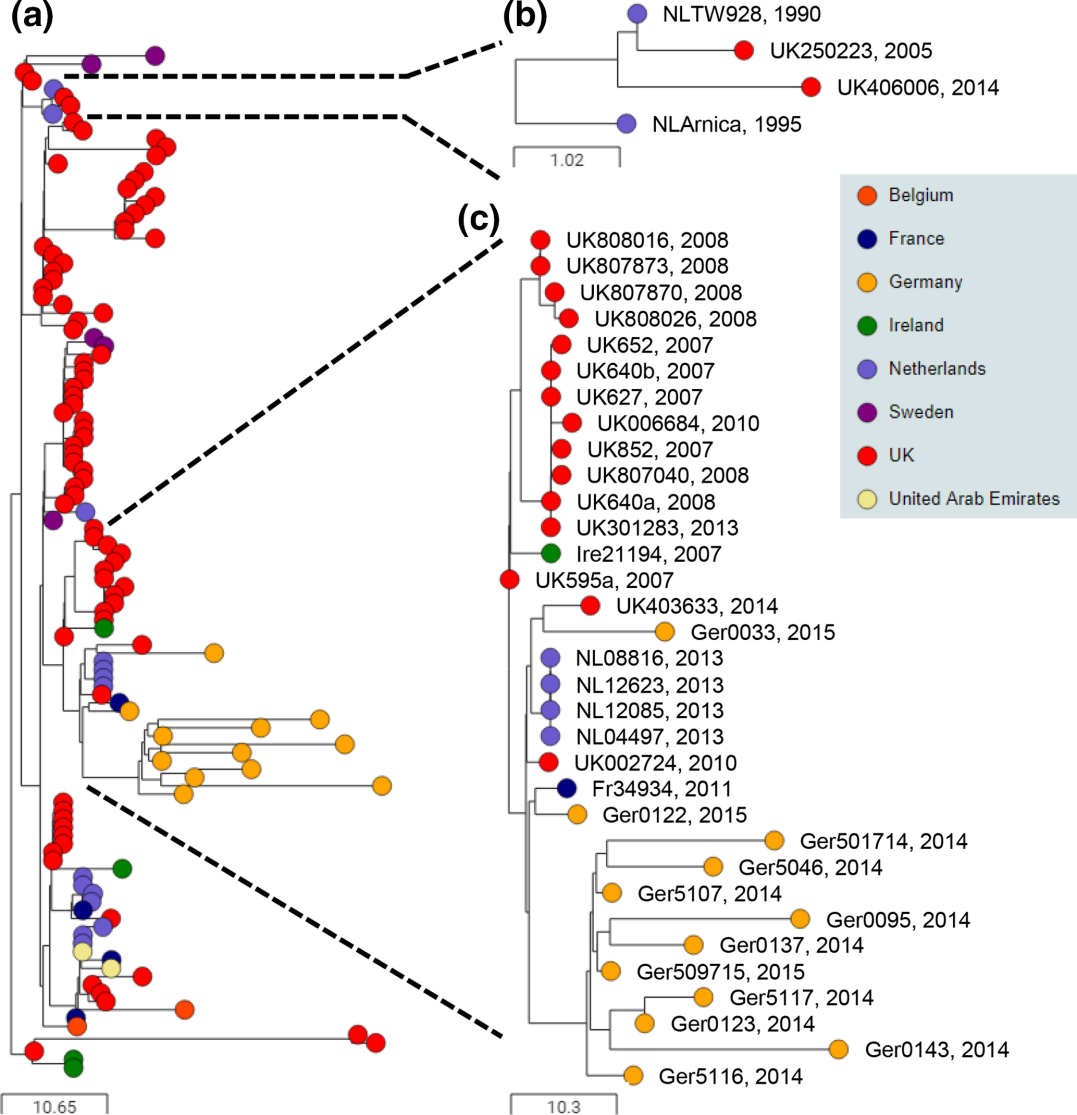
BAPS-5 is a European cluster that illustrates distant and recent introductions of *S. equi *. Midpoint-rooted phylogenetic reconstruction of the *S. equi* population visualized in Microreact. The dendrogram was reconstructed from pairwise cgMLST scores using the ape package [25]. The resulting tree was midpoint rooted using the phangorn package [26]. The scale bars relate to horizontal branch lengths and indicate the number of cgSNPs that are proposed to have occurred on the horizontal branches. Coloured circles indicate the country from which the isolates originated, as indicated in the key. (**a**) The phylogenetic relationships of the 126 isolates in the BAPS-5 subtree. (b) The Equilis StrepE subtree. (**c**) An example of a diverse outbreak strain from Germany and a closely related outbreak strain from the Netherlands. Isolate names and year of isolation are shown.

The 123 isolates in BAPS-5 were recovered from the UK (*n*=80), the Netherlands (*n*=14), Germany (*n*=12), Sweden (*n*=5), France (*n*=4), Ireland (*n*=4), UAE (*n*=2) and Belgium (*n*=2) (Fig. 6a). European isolates accounted for 98 % of isolates in BAPS-5. However, examination of the recovery of BAPS-5 strains of *S. equi* from horses in the UK identified a statistically significant decline in the prevalence of BAPS-5 strains, with 50 (36 %) of 137 isolates recovered from horses pre-2010 and 30 (18 %) of 170 isolates recovered between 2010 and 2016 (*P*=0.0002).

BAPS-5 includes the TW928 strain (named NLTW928 in Pathogenwatch) that is used in the Equilis StrepE live attenuated vaccine (MSD) (Fig. 6b). TW928 lacks *aroA* [35] and is administered via subcutaneous injection into the upper lip. The Arnica strain, which was used as a challenge during vaccine trials, clustered immediately next to strain TW928, differing by two pairwise cgSNPs. Isolates from two horses in the UK, UK250223 (Berwickshire) and UK406006 (Highland) that developed an adverse reaction and clinical signs of strangles, respectively, shortly after vaccination with Equilis StrepE, also clustered with these strains, differing from one another by between one and four pairwise cgSNPs across the core genome and containing the *aroA* deletion present in strain TW928 (Fig. 6b, Table S2).

All 12 of the isolates recovered from outbreak Ger-1 in the Lewitz area of Germany clustered into BAPS-5. All nine of the isolates recovered from horses during 2014 and one of the 2015 isolates, Ger5107, clustered together. However, two of the three isolates recovered in 2015, Ger0033 and Ger0122, were identified as being more closely related to strains recovered from horses in the UK and France (Fig. 6c). Strangles was known to have occurred on an annual basis in weanlings at the yard in Germany for several years, although the disease was typically of low severity [36]. Despite the relatively short time period over which these isolates were collected (November 2014 to March 2015), the genomes differed from one another by a mean of 21 pairwise cgSNPs (range 2 to 45). The mean substitution rate per core-genome site per year within *S. equi* was calculated as 5.22×10^7^ [10], equating to approximately one substitution per year within the core genome. Therefore, assuming variation accumulated evenly between strain pairs over time, these data suggest that the original strain of *S. equi* entered the horse population at Lewitz over 10 years previously. In contrast, the four isolates that were recovered on the 26th February 2010 from outbreak NL-3 near Klijndiik in the Netherlands that clustered within this branch of the BAPS-5 tree shared identical alleles across the core genome, suggesting a recent incursion of *S. equi* into this population of horses.

### BAPS-6 is a genetic lineage with limited prevalence

**Fig. 7.**
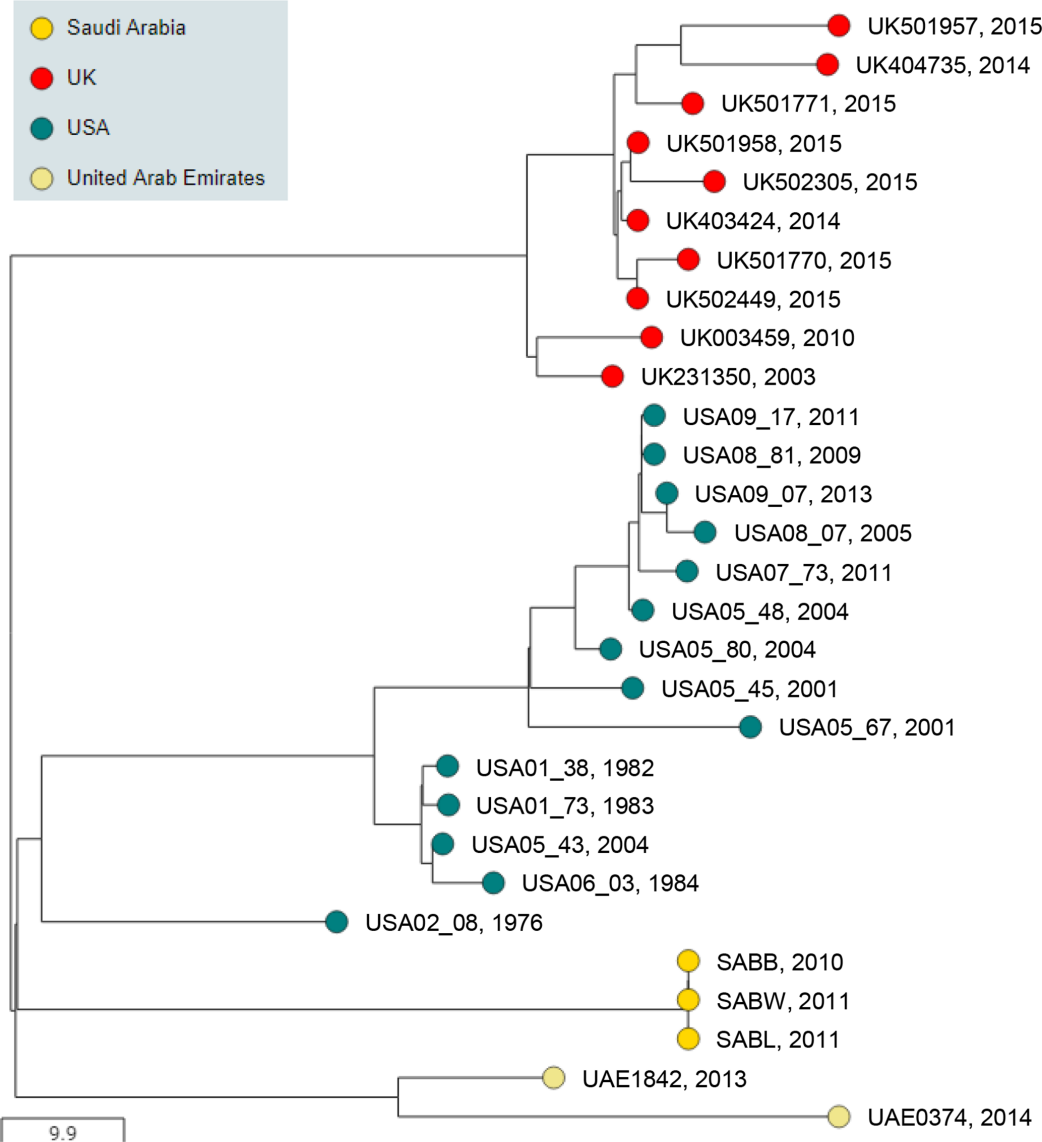
BAPS-6 contains geographically restricted sub-groups in the USA, UK, Saudi Arabia and UAE. Midpoint-rooted phylogenetic reconstruction of the *S. equi* population visualized in Microreact. The dendrogram was reconstructed from pairwise cgMLST scores using the ape package [25]. The resulting tree was midpoint rooted using the phangorn package [26]. The scale bar relates to horizontal branch lengths and indicates the number of cgSNPs that are proposed to have occurred on the branches. Coloured circles indicate the country from which the isolates originated, as indicated in the key. Isolate name and year of isolation are shown.

The 29 isolates that clustered together in BAPS-6 grouped into USA, UK, Saudi Arabia and UAE sub-groups that contained 14, 10, 3 and 2 isolates, respectively (Fig. 7). Although BAPS cluster 6 contained the fewest isolates, this cluster had the highest level of intra-cluster diversity, suggesting that it may subsequently split into multiple clusters as the collection of *S. equi* genomes increases. Five of the ten isolates from horses in the UK were recovered from an outbreak of strangles at the main yard of a horse sanctuary in Norfolk (UK-102) that began on the 20th February 2015 and affected 28 horses. Outbreak UK-102 was initially thought to have been caused by an unknown persistently infected carrier within a group of mares and foals, which had recovered from strangles in June 2014 (outbreak UK-103). The recovered mares and foals had been moved onto the main yard at the sanctuary just before the index case of UK-102 was identified, but the two isolates of *S. equi* (UK403660 and UK405405) that were recovered from UK-103 clustered into BAPS-2. Instead, BAPS-6 contained three other isolates (UK403424, UK404735 and UK501958) that had been recovered from horses at the sanctuary’s isolation facility between April 2014 and February 2015, identifying the source of outbreak UK-102 as the sanctuary’s isolation facility.

## Discussion

The horse is widely transited across the world, with over 150 000 horse trade events between European Union member states registered in 2016 alone [https://ec.europa.eu/food/sites/food/files/animals/docs/ahsc_report_2016_en.pdf (last accessed 22nd July 2020)]. To minimize the risks of diseases such as shipping fever, where the prolonged transport of horses via rail or sea can lead to respiratory disease and, occasionally, the death of the affected animal, horses are transported long distances by air [11]. However, the rapid movement of horses provides new opportunities for the transmission of infectious diseases. To minimize the risk of pathogen transmission, horses are examined by veterinarians pre- and post-transport. Whilst veterinary examinations identify animals suffering from acute disease, such as equine influenza, *S. equi* can persist in healthy animals that have recovered from strangles [5]. These carrier animals shed *S. equi* intermittently, and their national and international transportation can facilitate onward transmission to new populations of horses.

The identification of specific transmission events between horses, yards and countries has been hampered by the limited capacity of the conventional *S. equi* strain-typing methodologies to resolve population differences. The analysis of our collection of 670 isolates by MLST showed that each isolate possessed one of six closely related STs that are part of CC-179 [12]. A total of 198 SeM alleles have been identified in the online database [https://pubmlst.org/szooepidemicus/ (last accessed 22nd July 2020)], of which 103 SeM-types were represented within the 670 genomes examined here. However, despite this variability, 223 isolates contained the SeM-9 allele and a further 149 isolates encoded one of five other SeM alleles (SeM-6, 7, 28, 59 or 100). The dominant SeM-9 allele within BAPS-2 encoded consensus amino acid residues except for the BAPS-2-restricted amino acid variants Ser^62^ to Asp and Ala^127^ to Thr. The SeM-28 allele similarly encoded consensus amino acids at all sites except for Ser^143^ to Arg, which was present in 24 of 25 SeM alleles within BAPS-1. Our data suggest that the variability of this region of SeM is likely to be constrained by the functionality of the SeM protein. Therefore, whilst mutations and deletions occur, possibly in order to evade host immune responses during persistent infection [10, 20], we propose that some SeM variants are fitter than others and are more capable of onward transmission to naïve animals. It will be important to determine whether specific amino acid changes, such as those affecting Glu^58^, influence the functional activity of SeM and the fitness of *S. equi *.

Implementation of a significantly expanded cgMLST resource in Pathogenwatch, which sampled variation across 1286 alleles rather than the 7 alleles sampled by conventional MLST, separated the *S. equi* population into six BAPS clusters. Analysis of the BAPS clusters revealed statistically significant geographical associations with specific horse populations from around the world. BAPS-1 strains were associated with the USA, Israel and Japan; BAPS-2 with Europe and the UAE; BAPS-3 with Australasia; BAPS-4 with Argentina; and BAPS-5 with Europe. Examples of the recovery of genetically related isolates of *S. equi* from geographically distinct regions were identified across almost every BAPS cluster. Such potential international transmission events included the identification of two UK isolates, UK806841 and UK237364, in the BAPS-1 cluster, which are closely related to isolates of *S. equi* from the USA. Isolates from a series of strangles outbreaks in Israel, Japan and the UAE also clustered into BAPS-1, suggesting a link to the USA. Indeed, isolate Jpn0092 was recovered from a horse that developed acute disease shortly after import from the USA and whilst still in quarantine at the Shiroi facility in Chiba, Japan [13, 34]. The majority of isolates from Australia and New Zealand clustered together in BAPS-3, in keeping with their relative geographical isolation and trade. However, the presence of isolates UAE6134 and UAE6047 within this cluster provided a further illustration of the wider movement of horse populations and the associated risk of disease transmission from one country to another.

The cgMLST bioresource also provided sufficient resolution of the genetic distances between strains to allow the identification of potential transmission events at a national level. For example, the isolates recovered from outbreaks UAE-11, UAE-12 and UAE-13, which took place between January and June of 2014, clustered together. These isolates differed by a mean of eight pairwise cgSNPs and were distributed throughout this branch of BAPS-2 (Fig. 3b), suggesting that the outbreaks were interlinked. The recovery of *S. equi* from outbreak UAE-13 over 1 month after the recovery of similar strains from outbreaks UAE-11 and UAE-12 suggests that there may be an opportunity to identify a transmission point between these yards and possibly thereby prevent future outbreaks. Interestingly, horses in outbreak UAE-11 were infected with another three *S. equi* strains that clustered into BAPS-1, BAPS-2 and BAPS-6, demonstrating that this yard had suffered from incursions of four different strains of *S. equi* in just a 7 month period between November 2013 and June 2014.

The perception of inevitability, that all horses must suffer from strangles at some point in their lifetime, can be traced back to the 1600s [37]. However, strangles is not an inevitable rite of passage for horses [2]. Healthy horses that have been exposed to *S. equi* can be identified using a dual antigen iELISA (indirect ELISA) serological test [38]. The screening of biological samples taken from the guttural pouches or nasopharynx of horses exposed to, or suspected of being infected with, *S. equi* by quantitative PCR significantly improves the ability to identify persistently infected animals [39, 40], facilitating treatment in order to prevent onward transmission. Following examination of the initial analysis of the core-genome sequences presented here, the UAE introduced pre-import screening of all horses using the dual antigen iELISA as a mandatory requirement. This prompt and proactive response is believed to have already prevented countless new cases of disease through the identification and treatment of carriers to resolve their persistent infection prior to transportation (David Craig, personal communication).

The Pinnacle IN vaccine strains clustered within BAPS-1, whilst the TW928 strain used in the Equilis StrepE vaccine clustered within BAPS-5 alongside the Arnica strain that was used to measure the efficacy of this vaccine [35]. Two pairs of isolates were closely related to each of these vaccine strains, illustrating that cgMLST is a valuable tool for the identification of adverse reactions or cases of disease associated with live attenuated vaccines. A new multicomponent subunit vaccine based on the BAPS-2 isolate Sw1866 conferred up to 94 % protection against the BAPS-5 *Se*4047 strain (UK4047 in Pathogenwatch) [41]. BAPS-2 is now the most frequently isolated lineage of *S. equi* within Europe and the Middle East. The cgMLST bioresource within Pathogenwatch provides an opportunity to monitor the effects of vaccination on the population structure of *S. equi *, informing the design of future vaccines that account for the emergence, or introduction, of new strains from around the world.

The data presented here provide evidence of the international transmission of *S. equi* through horse populations in accordance with criterion one for the listing of terrestrial animal diseases by the OIE. It is already established that, in accordance with criterion 2, horses in Iceland remain free of *S. equi *, as a result of a ban on the import of horses, which has been maintained for over 1000 years [3]. The case definition of pyrexia and the formation of abscesses in the lymph nodes of the head and neck is well described, with the disease being first reported by Giordano Ruffo, farrier to Emperor Frederick II, in 1256 [42]. The causative agent, *S. equi *, was first identified in 1888 [8] and can be differentiated from the genetically related organism *S. equi* subspecies *zooepidemicus* by fermentation [22] or PCR assays [39, 40]. Therefore, it is possible to clearly identify cases and distinguish them from other diseases or infections in accordance with criterion 3 of the OIE. Finally, strangles has a significant impact on the health of horses, and the disease is reportable in the USA and in many other countries. Every horse at an affected premise can suffer from the disease and the mortality rate can reach 10 % in some outbreaks [43]. Therefore, strangles has a significant impact on the health of horses in accordance with criterion 4 of the OIE listing of terrestrial animal diseases. The increased prevalence of BAPS-2 strains in the UK since 2010, coupled with the recovery of BAPS-2 isolates from Europe, the Middle East and the USA, suggests that this cluster may already have achieved pandemic status. We urge the OIE to include strangles on the list of infectious diseases of Equidae of international importance. Such policy change will encourage intervention measures that will rein-in the onward transmission of *S. equi *, yielding significant health, welfare and economic benefits to the equestrian industry throughout the world.

The Pathogenwatch web-based cgMLST bioresource is available for the conduct of genomic surveys of both *S. equi* and its close relative *S. equi* subspecies *zooepidemicus *, shedding new light on the transmission dynamics and disease potential of these pathogens in horses and other animals, including humans.

## Supplementary Data

Supplementary material 1

Supplementary material 2
