# Supplementary material for: Globetrotting strangles: the unbridled national and international transmission of Streptococcus equi between horses
Source: Microb Genom. 2021 Mar 8;7(3):mgen000528. doi: 10.1099/mgen.0.000528 (PMC8190609; doi:10.1099/mgen.0.000528)
Supplement: Supplementary material 1 [file mgen-7-0528-s002.pdf]

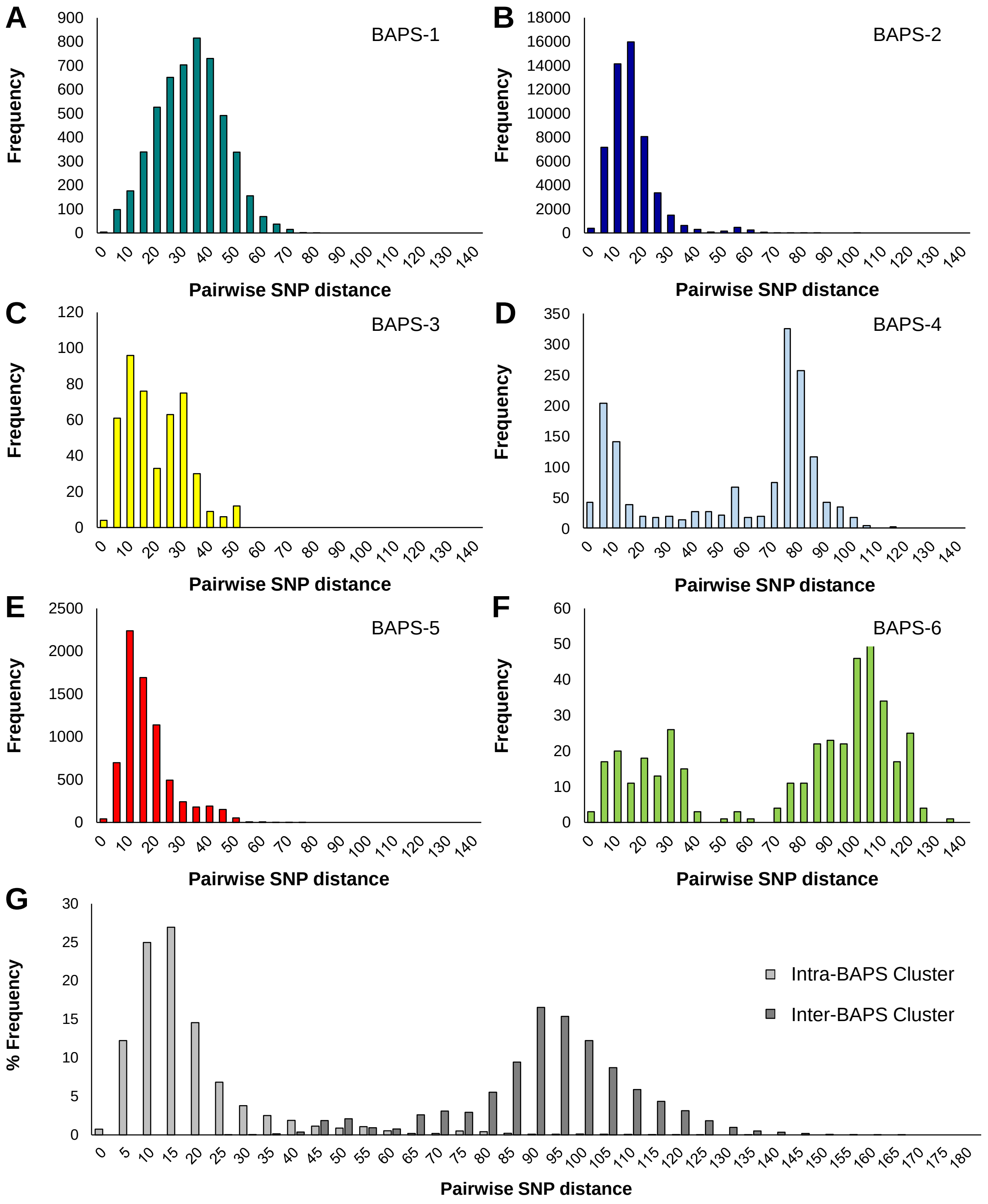

Fig. S1: Comparison of pairwise SNPs distances within and between BAPS clusters. Pairwise SNP distances between core genomes within (*A*) BAPS-1, (*B*) BAPS-2, (*C*) BAPS-3, (*D*) BAPS-4, (*E*) BAPS-5 and (*F*) BAPS-6. (*G*) Combined pairwise cgSNP distances between genomes within the six BAPS clusters (intra-BAPS in light grey) and between BAPS clusters (inter-BAPS in dark grey).
